# Adolescent alcohol exposure disrupts extinction learning and retrosplenial cortex physiology in adult males

**DOI:** 10.1101/2025.09.29.679287

**Published:** 2025-10-01

**Authors:** Lisa R. Taxier, Lili S. Kooyman, Sloan Y. Markowitz, Jobe L. Ritchie, Jessica A. Wojick, Thomas L. Kash

**Affiliations:** 1 Bowles Center for Alcohol Studies, University of North Carolina School of Medicine, Chapel Hill, North Carolina 27599; 2 Bowles Center for Alcohol Studies, University of North Carolina School of Medicine, Chapel Hill, North Carolina 27599; Department of Pharmacology, University of North Carolina School of Medicine, Chapel Hill, North Carolina 27599.

## Abstract

Adolescent binge drinking can lead to a myriad of issues later in life, including co-occurring diagnoses of Alcohol Use Disorder (AUD) and affective disorders such as post-traumatic stress disorder (PTSD). Although efforts to understand the effects of adolescent alcohol exposure on later health outcomes have unveiled lasting, maladaptive behaviors in adulthood, questions remain about region and cell-type specific mechanisms that drive such effects. Here, we tested the hypothesis that adolescent alcohol exposure would produce lasting alterations in retrosplenial cortex (RSC) function and physiology. In support of this hypothesis, we found that adolescent intermittent ethanol (AIE) vapor exposure resulted in impaired extinction recall of a trace fear memory in adulthood, as well as lasting reductions in intrinsic excitability in adult RSC pyramidal cells. Importantly, these changes were sex-specific, occurring in males but not females. Together, this work suggests that the RSC may be a key, vulnerable locus to adolescent alcohol’s detrimental effects.

## Introduction

Initiation of binge drinking during adolescence is one of the single greatest predictors of an Alcohol Use Disorder (AUD) and comorbid affective disorders such as post-traumatic stress disorder (PTSD) in adulthood[1,2]. Concerningly, comorbidity of AUD with PTSD in adulthood is associated with increased symptom severity and worse prognosis than either condition alone, underscoring the urgent need to develop better treatment strategies[3-5]. Cognitive dysfunction, which is exacerbated by adolescent drinking, is a common feature in adult patients with both AUD and PTSD[3-5]. However, the mechanisms through which adolescent binge drinking drives maladaptive cognition, particularly among associative learning processes, in adulthood remain largely uncharacterized. Therefore, advancing our understanding of how adolescent alcohol exposure produces persistent changes in adult brain function and behavior is critical for identifying novel therapeutic targets.

Effects of adolescent alcohol exposure on adult cognition have been well documented, with a particular focus on vulnerability of the hippocampus[6,7] and prefrontal cortex (PFC)[8-11]. However, the retrosplenial cortex (RSC), which is reciprocally connected to the hippocampus and PFC[12], has received comparatively little attention. This is somewhat surprising given the critical role that RSC plays in associative learning processes, including in contextual and spatial memory consolidation, retrieval, and extinction[12-16]. Emerging evidence, particularly from neuroimaging studies, implicates the RSC as a structure that may be vulnerable to adolescent alcohol exposure. For instance, functional connectivity of the RSC is modulated by alcohol exposure in humans[17] and mice[18]. In adults with AUD, the RSC is active in response to alcohol-associated cues[19,20]. This finding is mirrored in adolescents with AUD, suggesting that the RSC responds to alcohol during brain maturation[21]. Adult rats that underwent adolescent intermittent ethanol treatment exhibit increased cortical thickness of RSC[22]; yet, the functional relevance of this lasting change in morphology is unknown. Indeed, no work has been done to explore functional changes at a cellular level within the RSC that result from adolescent alcohol exposure.

One strategy to examine how adolescent alcohol exposure impacts the adult RSC, thereby influencing adult cognition, is to employ behavioral tasks in rodents that require RSC function. Importantly, the RSC is critical for acquisition and extinction of trace fear, a paradigm that requires attentional linking of the conditional stimulus (CS; tone) and the unconditional stimulus (UCS; footshock) across a stimulus-free temporal gap (the trace interval)[15,16]. Trace fear conditioning (TFC) is ideally suited for examination of complex memory formation and extinction, which is particularly important given that anxiety disorders including PTSD, which feature extinction impairments, are often treated via extinction-based exposure therapy. Some concern exists that exposure-based therapy may worsen risk for substance use relapse among individuals with AUD[23]. However, emerging evidence suggests that exposure therapy in combination with cognitive behavioral interventions can significantly reduce both AUD and PTSD symptom severity without increasing risk for relapse[24], underscoring that a mechanistic understanding of the interplay between alcohol exposure and extinction impairments may further advance this promising treatment strategy.

Here, we explored whether binge-like exposure to alcohol during adolescence via the adolescent intermittent ethanol (AIE) vapor model alters extinction learning and leads to disrupted RSC physiology in adulthood. Our results show that in male, but not female mice, AIE produces persistent effects on behavior and physiology. In particular, we observed a lasting deficit in extinction recall of a trace fear memory, and diminished intrinsic excitability of RSC pyramidal neurons in adult male, but not female mice exposed to AIE. Combined, these data implicate the RSC as a novel site for persistent effects of adolescent alcohol and provide insight into mechanisms of AIE-induced dysfunction of extinction behavior.

## Methods

### Animals

Male and female C57Bl/6J mice (n = 72); Stock #: 000664, Jackson Laboratories) were obtained from Jackson Laboratories at postnatal day 21 (P21). Mice were group-housed 4/cage and maintained on a 12-hour reverse light-dark cycle in temperature- and humidity-controlled facilities, with *ad libitum* access to food (Prolab Isopro RMH 3000, LabDiet) and water. All procedures followed the National Institutes of Health Guide for the Care and Use of Laboratory Animals and were approved by the University of North Carolina-Chapel Hill School of Medicine Institutional Animal Care and Use Committee.

### Adolescent Intermittent Ethanol

Mice were exposed to air or AIE via air or ethanol vapor chamber on a 2 days-on, 2 days-off schedule, for 16 hrs/day beginning at P28, and ending at P60. Vapor chambers were calibrated to produce average blood ethanol concentrations (BECs) meeting the National Institute on Alcohol Abuse and Alcoholism definition of binge alcohol exposure (0.08%, or 0.08 grams of alcohol per deciliter or higher). Tail blood was taken at 3 interspersed intervals throughout the duration of AIE, and BECs were measured using an Analox-AM1 (Analox Technologies).

### Trace Fear Conditioning

Trace fear conditioning, extinction, and extinction recall were conducted across four days. On the first day, mice were given one 5-minute context pre-exposure session in Context A (grid floor, cleaned with a 20% ethanol + 1% vanilla solution), during which they were allowed to freely explore the context. On day 2 (conditioning), mice were placed into Context A; following a 2 min baseline, mice were subjected to 5 tone-shock pairings separated by a 20 s stimulus-free trace interval (tone: 20 s, 80 dB, 3KHz; trace: 20 s; shock: 0.6 mA, 2 s). Each tone-trace-shock presentation was separated by a 120 s inter-trial interval. On day 3 (extinction), mice were placed into a novel context B (curved walls, white plastic flooring, cleaned with 0.5% acetic acid). After a 2 min baseline, mice were presented with 20 tone presentations separated by a 60 s inter-trial interval. On day 4 (extinction recall), mice were again placed into context B and received tone/ITI presentation as in day 3. Fear box (Med Associates) hardware was controlled by Ethovision XT (Noldus).

### Slice electrophysiology

Whole-cell patch-clamp recordings were obtained from pyramidal cells residing in layer 5/6 of the anterior granular RSC (within −1.06 to −1.7 AP in stereotaxic coordinates). Following rapid decapitation, brains were extracted and immersed in a chilled sucrose cutting solution (194 mM sucrose, 20 mM NaCl, 4.4 mM KCl, 2 mM CaCl_2_, 1 mM MgCl_2_, 1.2 mM NaH_2_PO_4_, 10 mM d-glucose, and 26 mM NaHCO) oxygenated with 95% O_2_/5% CO_2_. Coronal sections (250 μM) containing the RSC were obtained using a Compresstome VF-510-OZ (Precisionary Instruments) and allowed to recover for 30 minutes in 35°C oxygenated artificial cerebrospinal fluid (aCSF; 124 mM NaCl, 4.4 mM KCl, 1 mM NaH2PO4, 1.2 mM MgSO4, 10 mM D-glucose, 2 mM CaCl2, and 26 mM NaHCO3) prior to the start of recording. A cesium methanesulfonate-based internal solution (135 mM cesium methanesulfonate, 10 mM KCl, 1 mM MgCl2, 0.2 mM EGTA, 4 mM MgATP, 0.3 mM Na2GTP, 20 mM phosphocreatine, with 1mg/mL QX-314) was used during recordings of spontaneous inhibitory and excitatory post-synaptic currents (sIPSCs and sEPSCs), and a potassium gluconate-based internal solution (135 mM K-gluconate, 5 mM NaCl, 2 mM MgCl2, 10 mM HEPES, 0.6 mM EGTA, 4 mM Na2ATP, 0.4 mM Na2GTP) was used for intrinsic excitability experiments. Recording pipettes (2–4 MΩ) were pulled from thin-walled borosilicate glass capillaries using a P95 pipette puller (Sutter Instruments). Signals were acquired using an Axon Multiclamp 700B amplifier (Molecular Devices), digitized at 10 kHz, filtered at 3 kHz, and analyzed in Easy Electrophysiology. Series resistance (*R*_s_) was monitored without compensation, and changes in *R*_s_ exceeding 20% were used as exclusion criteria.

### Synaptic input mapping

Slices were prepared as described above. Synaptic input mapping was conducted with Laser Assisted Stimulation and Uncaging software (LASU, Scientifica) as previously published [25]. 35 mL RuBi-glutamate (300 μM) in aCSF was oxygenated and recirculated, and was photolysed using a pulsed 405 nm laser beam (50 mw) at 5x magnification across a stimulus grid (200 μM spacing) placed across the RSC, including all cortical layers. Uncaging at each stimulation site was guided by mirror galvanometers controlled by LASU software, and was separated by 5s intervals. To isolate IPSCs and EPSCs, maps were recorded at a holding potential of +10 mV or −55 mV, respectively, and a cesium methanesulfonate internal solution (135 mM cesium methanesulfonate, 10 mM KCl, 1 mM MgCl2, 0.2 mM EGTA, 4 mM MgATP, 0.3 mM Na2GTP, 20 mM phosphocreatine, with 1mg/mL QX-314) was used. IPSCs were observed within a 2–50 ms window after the stimulus for each sweep, whereas EPSCs were observed within a 3-25 ms window (Brill et al, 2016). Amplitude of each uncaging-evoked event was recorded in pClamp 10.7, and individual synaptic input maps were combined to generate averaged synaptic input maps by recording the amplitude, in pA, and x and y distance, in microns, of each responsive stimulation site from the soma. These values were then binned at 200 μm intervals. Smooth contours were derived in MATLAB using linear interpolation between 200 μm bins.

### Statistical analyses

Statistical analyses were performed using GraphPad Prism 10 software or SPSS (IBM). Behavioral data were analyzed using three-way ANOVAs using sex (male vs female), treatment (Air vs AIE), and stimulus (either tone presentation across time for acquisition, or BL, CS, and ITI) as independent variables. For analyses where the sexes were analyzed separately, unpaired *t* tests were used to compare total number of synaptic input sites, rheobase, action potential (AP) kinetic metrics, and differences in amplitude/frequency of spontaneous excitatory or inhibitory currents.

Two-way ANOVAs using treatment (Air vs AIE) and distance to soma as independent variables were used for synaptic input mapping experiments to compare summed cumulative synaptic inputs. Significant ANOVA interactions were followed by Šídák’s multiple-comparisons test, with significance set at *p* < 0.05.

## Results

### AIE impairs extinction recall in adult males but not females

**Figure 1.**
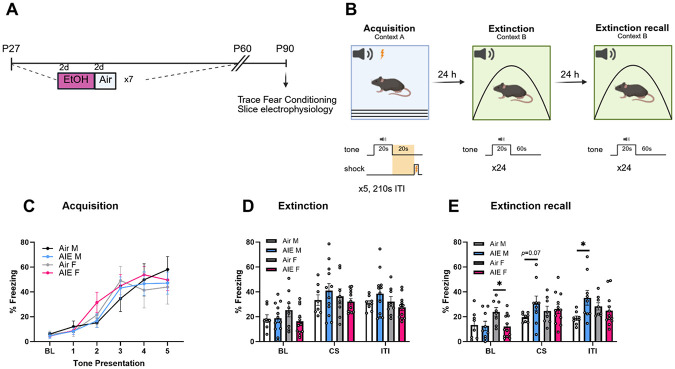
AIE impairs extinction recall in adult males but not females. (A) Experimental Timeline. Mice were exposed to air or ethanol vapor for 16 hr/day on a 2-days-on, 2-days-off schedule from P27 through P60 prior to trace fear conditioning and slice electrophysiology (separate cohorts) at P90. (B) Trace fear conditioning procedure. (C) males and females increased freezing from baseline (BL) across consecutive tone presentations. (D) During extinction, there were no AIE-mediated changes in freezing behavior during BL, CS presentations, or ITIs. (E) During extinction recall, Air-exposed females froze more at baseline than AIE-exposed females, and AIE-exposed males froze more during CS presentations and ITIs than Air-exposed males. *p <0.05, error bars indicate SEM.

We first aimed to determine whether AIE produces long-lasting effects on trace fear learning in adulthood. To accomplish this, we subjected mice to air or AIE from P28-P60, followed by trace fear conditioning at P90 (Fig. 1A, B). We found that both male and female mice exposed to AIE readily acquired fear (Fig. 1C; three-way ANOVA, *F*_(5, 180)_ = 27, *p* < 0.001), but no significant effect of AIE. Both males and females extinguished fear on extinction day 1 regardless of AIE; there was a significant effect of stimulus (Fig. 1D; three-way ANOVA, *F*_(2, 72)_ = 34.13, *p* < 0.001). However, on extinction recall day, males exposed to AIE continued to freeze to a higher extent relative to air controls, as revealed by a significant sex x treatment interaction (Fig 1E, *F*_(1,33)_ = 4.44, *p* = 0.04). Sidak’s post hoc comparisons revealed a trend towards higher freezing during CS presentations in AIE-exposed males compared to Air controls (*p* = 0.07), and significantly higher freezing during the ITI in AIE-exposed males compared to Air controls (*p* = 0.01). Although Air-exposed females froze more than AIE-exposed females at baseline during extinction recall (*p* = 0.02), there were no AIE-mediated differences in freezing during CS or ITI presentations in females, suggesting that the effect of AIE on extinction recall was sex-specific.

### AIE produces diminished intrinsic excitability in adult RSC of males, but not females

**Figure 2.**
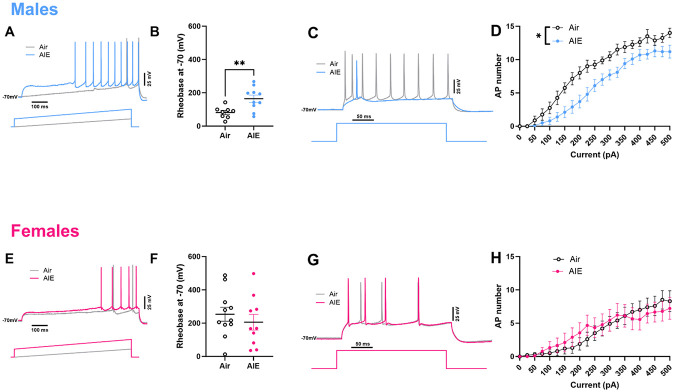
AIE diminishes RSC pyramidal cell intrinsic excitability in adult males, but not females. Representative traces from males (A) and females (E) show examples of increased rheobase in cells from AIE-exposed males (B) but no AIE-mediated effects on rheobase in females (F). Representative traces from males (C) and females (G) show examples of current-induced firing, which is reduced in cells from AIE-exposed males (D), but not in females (H). * p < 0.05, ** p <0.01, error bars indicate SEM.

We next examined whether the excitability of cells within the adult RSC, a region critical for trace fear extinction, was altered by AIE. As before, we exposed mice to air or AIE from P28-P60, and allowed mice to age alcohol-free until P90, when we collected slices containing the anterior RSC for whole-cell patch-clamp electrophysiology. Because we observed no effect of AIE in females, we chose to analyze the sexes separately. In males that underwent AIE, rheobase, or minimum amount of current required for an action potential to fire, was significantly higher compared to air controls (Fig 2A and B; *t*_(16)_ = 3.07, *p* < 0.01). This effect was sex-specific, as rheobase in females was unaltered following AIE (Fig 2E and F; *t*_(19)_ = 0.76, *p* = 0.46). We next examined action potentials fired in response to increasing stepwise current injections, and found that in AIE-exposed males, cells fired fewer action potentials in response to increased current injections compared to air controls (Fig2C and D; two-way repeated measures ANOVA, *F*_(1,16)_ = 8.58, AIE: *p* <0.01). There was also a significant interaction between current step and treatment (*F*_(20,320)_ = 3.07, *p* < 0.0001). As with rheobase, this effect of AIE was sex-specific; there were no differences in action potentials fired in response to increasing current injections in female AIE-exposed adults compared to air controls (Fig 2E and F; *F*_(1, 17)_ = 0.02, *p* = 0.89), nor an interaction between current step and treatment (*F*_(20, 340)_ = 0.71, *p* = 0.81). Combined, these data suggest that AIE diminishes intrinsic excitability in adult male, but not adult female RSC.

**Figure 3.**
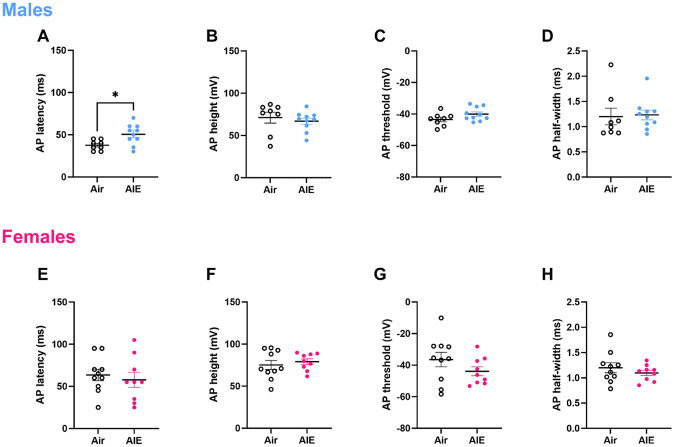
AIE increases AP latency in RSC pyramidal cells of adult males, but not females. Latency to fire an action potential is increased in RSC pyramidal cells from adult males (A) but not females (E). AP height (B, F), AP threshold (C, G) and AP half-width (D, H), were unchanged by AIE in both sexes. * p < 0.05, error bars indicate SEM.

Intrinsic excitability, which is a crucial regulator of learning and memory processes, can be modulated by changes in ion channel distribution and function[26]. To test the hypothesis that AIE exerts effects on intrinsic excitability via altering ion channel function, we assessed metrics of action potential (AP) kinetics including AP latency, height, threshold, and half-width. AIE increased AP latency in males (Fig 3A, *t*_(16)_ = 2.76, *p* = 0.01), but had no significant effect on AP height (Fig 3B), threshold (Fig 3C), or half-width (Fig 3D). Again, any observed effects of AIE on AP kinetics were sex-specific; AIE had no demonstrable effects on AP kinetics in adult female RSC (Fig 3E-H). Given that AIE increased AP latency in males, taken together with observed effects of AIE on intrinsic excitability, these data suggest that diminished intrinsic excitability may be due in part to AIE modulation of ion channel function within the adult male RSC.

### AIE reduces excitatory drive in adult RSC of males, but not females

**Figure 4.**
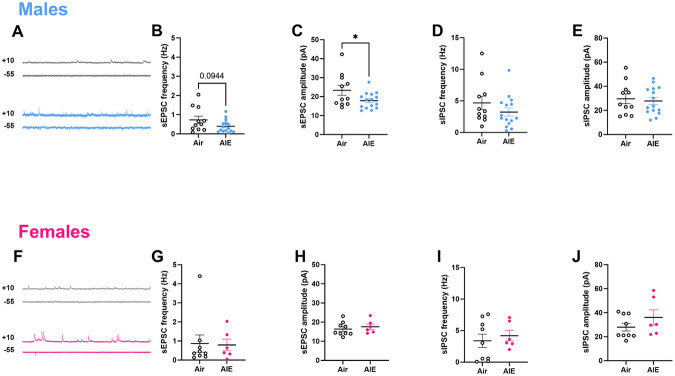
AIE reduces spontaneous excitatory drive in adult RSC of males, but not females. Representative recordings from Males (A) and Females (F) showing spontaneous firing of EPSCs recorded at −55 mV, or IPSCs recorded at +10 mV. sEPSC frequency (B) and amplitude (C) were reduced in cells from AIE-exposed males, but not females (G, H). sIPSC frequency (D,I) and amplitude (E,J) were unaffected by AIE exposure in either sex. *p <0.04, error bars indicate SEM.

To evaluate spontaneous synaptic transmission in adult RSC following AIE, we used a cesium methanesulfonate-based internal solution (see methods) to isolate sEPSCs and sIPSCs in the same cells while voltage clamping at −55 mV and +10 mV, respectively (Fig 4A, F). This approach allows for the evaluation of excitatory-inhibitory (E/I) drive onto individual neurons. Males displayed a trend towards AIE-diminished frequency of sEPSCs (unpaired *t*-test, *t*_(24)_ = 1.74, *p* = 0.09), and an impact of AIE on sEPSC amplitude such that RSC cells from AIE-exposed males had a significantly lower amplitude than those from air-exposed controls (*t*_(24)_ = 2.11, *p* = 0.05). There was no such effect of AIE on sIPSC frequency (Fig 4 D) or amplitude (Fig 4 E) in males. In females, there were no significant differences in frequency or amplitude of sEPSCs (Fig 4 G, H); this null effect was also present for both frequency and amplitude of sIPSCs (Fig 4. I,J).

### AIE reduces the number of local excitatory synaptic inputs to adult male RSC pyramidal neurons

**Figure 5.**
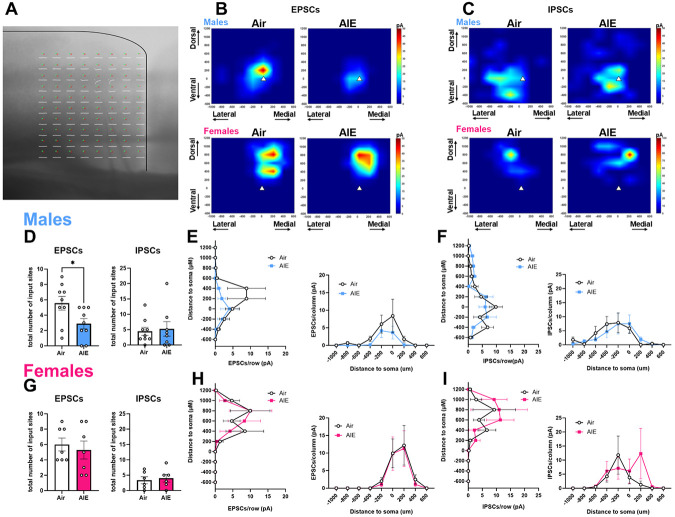
AIE reduces the number of local excitatory synaptic inputs to adult male RSC pyramidal neurons. (A) Representative grid superimposed over the extent of the left hemisphere of the anterior RSC, with corresponding traces in white detailing sEPSCs elicited by glutamate uncaging at each grid site (green crosses). (B) Representative summed input maps of sEPSCs elicited by glutamate uncaging in males (top) and females (bottom), in RSC pyramidal cells from both Air (left) and AIE (right)-exposed mice. (C) Representative summed input maps of sIPSCs elicited by glutamate uncaging in males (top) and females (bottom), in RSC pyramidal cells from both Air (left) and AIE (right)-exposed mice. (D) Total number of input sites where EPSCs, but not IPSCs were elicited was reduced cells from AIE-exposed mice compared to cells from air-exposed controls. (E) Amplitude of uncaging-evoked EPSCs tended to be lower onto cells from AIE-exposed males across both the inter- and intralaminar extent, whereas amplitude of uncaging-evoked IPSCs (F) was unaffected by AIE. (G) In females, there was no AIE-mediated difference in total number of input sites where EPSCs or IPSCs were evoked by glutamate uncaging, nor was there an effect of AIE on averaged inter- or intralaminar amplitude of evoked EPSCs (H) or IPSCs (I).

Excitatory neurons within the superficial layers of RSC, which receive robust local inhibitory input from neighboring interneurons, project through deeper layers of RSC and into the corpus callosum[27]. Diminished intrinsic excitability and reduced glutamatergic drive onto layer 5/6 pyramidal neurons of male RSC following AIE could potentially arise from decreased excitatory or increased inhibitory input from layer 2/3, or from fast spiking interneurons present across the RSC laminar extent. To examine whether local synaptic input might contribute to our observed effect of AIE on intrinsic excitability, we conducted synaptic input mapping experiments in adult RSC following AIE. We patched onto pyramidal cells in layer 5/6, and uncaged Rubi-glutamate using focal laser application at 200μM intervals across the extent of the RSC (Fig. 5A) while voltage clamping at −55 and +10 to isolate uncaging-evoked EPSCs and IPSCs, respectively. This approach allowed for the detection of the presence, strength, and spatial location of local synaptic excitatory and inhibitory inputs to patched cells, and we generated averaged synaptic input maps to all patched cells to visualize the spatial pattern and strength of such inputs (Fig. 5B and C). In males, the total number of sites where EPSCs, but not IPSCs, were evoked was reduced in cells from AIE-exposed mice relative to air-exposed controls (Fig. 5D, left; *t*_(16)_ = 2.34, *p* = 0.03). In line with this, when cumulative amplitude was collapsed across the interlaminar extent (i.e., horizontal distance to soma), two-way ANOVA revealed a trend towards an effect of AIE (Fig 5E, left; *F*_(1,160)_ = 2.85; *p* = 0.09), such that the amplitude of uncaging-elicited EPSCs was lower in cells from AIE-exposed males compared with air-exposed controls. Similarly, when cumulative amplitude was collapsed across the intralaminar extent (i.e., vertical distance to soma), two-way ANOVA revealed a trend towards an effect of AIE (Fig 5E, right; *F*_(1,162)_ = 3.02, *p* = 0.08), such that amplitude of uncaging-evoked EPSCs was lower in cells from AIE-exposed males compared with air-exposed controls. For IPSCs, there was no significant effect when cumulative amplitude was collapsed across either the intralaminar (Fig 5F, left) or the interlaminar (Fig 5F, right) extent. In females, as with every other examined metric, there was no significant effect of AIE on total input sites for EPSCs or IPSCs (Fig 5G), no significant effect of AIE on EPSC amplitude along the dorsal/ventral axis (Fig 5H, left) or medial/lateral axis (Fig 5H, right), and no significant effect of AIE on IPSCs amplitude along the dorsal/ventral axis (Fig 5I, left) or medial/lateral axis (Fig 5I, right). For all collapsed cumulative amplitude measures, regardless of sex, there was a significant effect of either dorsal/ventral or medial lateral distance such that amplitude of evoked EPSCs and IPSCs were higher at uncaging sites near the soma.

## Discussion

Although considerable evidence points to a role for adolescent alcohol in modulating adult cognitive behavior and brain function, questions remain about region specific mechanisms that drive such effects. Here, we provide novel evidence that the RSC is susceptible to adolescent alcohol exposure in a sex-specific manner. We show that recall of extinction following trace fear conditioning is impaired in adult male mice, but not females, exposed to AIE. Furthermore, we show that AIE produces lasting decreases in intrinsic excitability of pyramidal cells within the adult male, but not female RSC, and that such changes in intrinsic excitability are accompanied by increased action potential latency. We also demonstrate that AIE produces reductions in spontaneous excitatory drive onto adult male, but not female, RSC pyramidal cells, and that AIE produces diminished local excitatory synaptic input onto adult male, but not female RSC pyramidal cells.

Our finding that extinction recall is impaired following AIE in males adds to a complex literature demonstrating that exposure to alcohol during adolescence can alter aspects of fear learning in adulthood. Previous work examining the effects of adolescent alcohol exposure on fear learning in adulthood has produced mixed findings that make interpretation of adolescent-alcohol driven effects on fear behavior challenging. Adolescent alcohol exposure sped acquisition of delay fear[28,29], reduced freezing during retention[30] or extinction of delay fear [28], enhanced freezing during extinction recall [29], and impaired contextual fear extinction [31]. Furthermore, these findings varied by sex [28,29]. Any number of differences in methodology could explain such divergent effects; for example, different paradigms of exposure (e.g. voluntary or not; oral vs inhalation), strain or species, and/or timing of alcohol exposure may affect adult behavior differently. Another alternative explanation is that the neural mechanisms governing different types of fear behaviors (e.g. trace or contextual fear vs delay fear) are fundamentally different [32], and may therefore be affected in unique ways by alcohol exposure. Our findings are congruent with the possibility that adolescent alcohol may have a more pronounced effect on fear behaviors in which animals must encode either the spatial or temporal context, as is required for trace fear conditioning. To our knowledge, the current study is the first to demonstrate that adult males show a lasting detrimental impact of AIE on extinction recall following extinction of a trace fear memory. Modeling the formation and extinction of more complex fear memories using trace fear conditioning may be a useful tool for those continuing to study the interplay between alcohol history and cognitive-affective dysfunction in preclinical models.

One surprising result of the current experiments was that there was no detectable effect of AIE on trace fear conditioning nor RSC physiology in females. Although our work is consistent with previously published work showing male-specific effects of AIE on contextual fear conditioning[33], this still suggests the need for preclinical models that might better capture the detrimental effects of adolescent alcohol on adult behavior in both sexes. One possibility for lack of observed effect in females is the timing of exposure; although exposure across P28-P55 captures broadly agreed-upon definitions of the adolescent window[34], it may be the case that female mice may be more vulnerable to alcohol exposure at earlier or later time points than were investigated in the current study. Another possibility is that females are more resilient to the effects of alcohol exposure during the adolescent window than males, which presents an intriguing possibility that deserves additional exploration. Although we did not make a statistical comparison between sexes, we observed that females seem to exhibit a much less excitable phenotype at baseline compared to males. This observation highlights the need for future work to investigate baseline sex differences in electrophysiological phenotypes within the RSC. Additional behavioral assays should also be used in future studies so as to better capture the ways in which adolescent alcohol might impact PTSD-relevant behaviors in adult females; these could include novelty-induced hypophagia, which others have shown is sensitive to adolescent alcohol [35].

Our finding that adolescent alcohol robustly and persistently diminishes intrinsic excitability within the adult male RSC is particularly striking given studies demonstrating the opposite effect of adolescent alcohol on intrinsic excitability of pyramidal neurons within other structures such as the prelimbic cortex [9,36]. Furthermore, in the hippocampus, AIE facilitates low stimulus intensity-induced LTP [37], suggesting a shift towards AIE-driven excitation within this region. Although one popular theory posits that adolescent-like phenotypes favoring excitation over inhibition persist into adulthood following adolescent alcohol exposure, our data indicate that the RSC may be an exception to this idea [38]. However, it may also be the case that the RSC maintains a different profile of excitatory and inhibitory balance during adolescence than other cortical structures. Indeed, one study suggests that neurons within the RSC that exhibit an afterdepolarization following action potential firing do not emerge until later in development, which is notable given that this particular firing property seems to enhance excitability [39]. Although we did not examine intrinsic excitability in the adolescent RSC in the absence of alcohol exposure, future work should assess whether adolescent alcohol perpetuates existing adolescent-like phenotypes within this region.

Surprisingly, we found no effect of adolescent alcohol on spontaneous inhibitory drive or on local inhibitory synaptic input to adult RSC pyramidal cells. Because the RSC is populated with several interneuron subtypes including parvalbumin-expressing interneurons, vasoactive inhibitory peptide-expressing cells, and somatostatin-expressing interneurons, we suspected that diminished intrinsic excitability of pyramidal cells within adult RSC following AIE may be due to increased inhibition from these cell populations. However, given our synaptic input mapping findings and lack of effect of AIE on spontaneous inhibitory drive, we speculate that long-range inhibitory projections to RSC, such as those from the PFC or the hippocampus, may play a more consequential role in driving the effects of AIE on decreased intrinsic excitability. For example, a population of somatostatin neurons in the PFC project to the RSC[40] and excitability of prelimbic somatostatin neurons is increased following adolescent alcohol exposure[11]. This suggests the possibility that following AIE, long-range somatostatin projections from the PFC impinge upon RSC pyramidal cells to inhibit glutamatergic drive and diminish intrinsic excitability.

Taken together, our data demonstrate that binge-like exposure to alcohol during adolescence has lasting effects on fear extinction processes and physiological function of the adult male RSC. Specifically, AIE perpetuates freezing during extinction recall in males, but not females, suggesting impaired ability to extinguish a trace fear memory. Additionally, our data suggest that reduced excitability of pyramidal cells within the adult RSC may underlie this behavioral finding. These findings add critical information about how adolescent alcohol can shape adult brain function and behavior, and point towards a novel brain target for additional study. Given the dense interconnectedness of the RSC, future studies should examine the role of AIE on both inputs and outputs to RSC. Furthermore, future work could assess if manipulating RSC function of RSC in adulthood rescues AIE-induced deficits. Such research will ultimately add to mechanistic understanding of how AIE might produce lasting adult dysfunction and ultimately lead to the development of new therapeutics.
